# Bacterial molecular machinery in the Martian cryosphere conditions

**DOI:** 10.3389/fmicb.2023.1176582

**Published:** 2023-07-26

**Authors:** Víctor Muñoz-Hisado, Fátima Ruiz-Blas, Jesús Manuel Sobrado, Eva Garcia-Lopez, Emma Martinez-Alonso, Alberto Alcázar, Cristina Cid

**Affiliations:** ^1^Centro de Astrobiologia (CAB), CSIC-INTA, Madrid, Spain; ^2^GFZ German Research Centre for Geosciences, Section Geomicrobiology, Telegrafenberg, Potsdam, Germany; ^3^Hospital Ramón y Cajal, Instituto Ramón y Cajal de Investigación Sanitaria, Madrid, Spain

**Keywords:** Mars, microorganisms, *Bacillus subtilis*, *Curtobacterium flacumfaciens*, stress response, planetary protection, proteomics, MALDI-TOF MS

## Abstract

The exploration of Mars is one of the main objectives of space missions since the red planet is considered to be, or was in the past, potentially habitable. Although the surface of Mars is now dry and arid, abundant research suggests that water covered Mars billions of years ago. Recently, the existence of liquid water in subglacial lakes has been postulated below the South pole of Mars. Until now, experiments have been carried out on the survival of microorganisms in Martian surface conditions, but it remains unknown how their adaptation mechanisms would be in the Martian cryosphere. In this work, two bacterial species (*Bacillus subtilis* and *Curtobacterium flacumfaciens*) were subjected to a simulated Martian environment during 24 h using a planetary chamber. Afterward, the molecular machinery of both species was studied to investigate how they had been modified. Proteomes, the entire set of proteins expressed by each bacterium under Earth (named standard) conditions and Martian conditions, were compared using proteomic techniques. To establish this evaluation, both the expression levels of each protein, and the variation in their distribution within the different functional categories were considered. The results showed that these bacterial species followed a different strategy. The *Bacillus subtilis* resistance approach consisted of improving its stress response, membrane bioenergetics, degradation of biomolecules; and to a lesser extent, increasing its mobility and the formation of biofilms or resistance endospores. On the contrary, enduring strategy of *Curtobacterium flacumfaciens* comprised of strengthening the cell envelope, trying to protect cells from the extracellular environment. These results are especially important due to their implications for planetary protection, missions to Mars and sample return since contamination by microorganisms would invalidate the results of these investigations.

## 1. Introduction

The exploration of Mars is one of the main objectives of space missions since the red planet is considered to be potentially habitable. Although possible life on Mars may be different from life on Earth, the main conditions of habitability could be common. Several minimum conditions of habitability have been defined: a solvent (water), a source of energy, a group of biologically essential elements (on Earth they are H, C, N, O, S, and P), and some physicochemical characteristics (temperature, pH, water activity, etc.) (Cockell et al., 2016). Mars is a cold and dry planet, with temperatures ranging from −125°C to 20°C, with an atmosphere composed of 96% CO_2_ (the rest is mainly N and Ar), an average atmospheric pressure of 7 mbar and UVB and UVC radiation levels about 200–315 nm (~361 kJ/m^2^) (Garcia-Lopez and Cid, 2017; Cortesão et al., 2019). Several studies have determined that different microorganisms were resistant to the extreme environmental conditions such as radiation (Hansen et al., 2005, 2009), desiccation, temperature extremes and pressure (Baqué et al., 2013; Mastascusa et al., 2014), on the Martian surface. In addition, it should be taken into account that some elements present on the planet’s surface, such as dust or rocks, could protect microorganisms from exposure to these extreme conditions, thus lengthening their lifespan (Cortesão et al., 2019). It has also been determined that some areas on Mars would facilitate the survival of these microorganisms due to their temperature or humidity features (Rummel et al., 2014; Rettberg et al., 2016), and the possibility of life transfer from one planet to another by means of rocks (Scalzi et al., 2012).

Furthermore, although the surface of Mars is now dry and arid, abundant research proposes that water covered Mars billions of years ago. Frozen water has been suggested to exist, not just at high latitudes at the Martian poles, but also at mid-latitudes (Bramson et al., 2015; Morgan et al., 2021). And recently, the detection of liquid water by several exploration instruments such as the Mars Advanced Radar for Subsurface and Ionosphere Sounding (MARSIS) have revived the debate about the origin and stability of liquid water under present-day Martian conditions (Lauro et al., 2021; Arnold et al., 2022). By analogy with what happens on Earth, glacier beds reach the pressure melting point, and subglacial lakes in Mars would be the result of the frictional heat produced by flowing ice concentrated at the base of the ice mass, which warms the ice-bedrock interface (Arnold et al., 2022).

The presence of microorganisms in the terrestrial cryosphere and in subglacial lakes (Rogers et al., 2013), broadens the spectrum of places where life could be possible on Mars. In this work, to check whether terrestrial microorganisms were capable of withstanding and living in the Martian cryosphere, i.e., subglacial lakes, two different species of bacteria (*Bacillus subtilis* and *Curtobacterium flaccumfaciens*) were exposed to Martian conditions in a simulation chamber. Subsequently, their molecular machinery was studied by proteomic techniques to investigate both the presence and absence of certain proteins and their expression levels. Hitherto, studies have been reported on the survival of *Bacillus subtilis* in Martian conditions as a spore (Cortesão et al., 2019), as well as its survival in soils analogous to those of Mars (Schuerger et al., 2017), focusing on the damage that would occur in DNA. The resistance of some bacteria and fungi subjected to Martian environmental conditions has also been published (Cortesão et al., 2021). However, how the cellular machinery of these cells changes and adapts has not yet been investigated. With this work, we try to increase knowledge about the possibilities of adaptation of these bacteria to the Martian environments.

The results of these experiments show that the survival of bacteria in Martian conditions is one of the greatest risks for planetary protection, that is, the practice that is responsible for protecting the other bodies of the solar system from contamination by terrestrial living organisms. Extremophile microorganisms could spread to other celestial bodies where environmental conditions were favorable.

These experiments are intended to answer several questions: (i) Are terrestrial bacteria capable of withstanding Martian cryosphere conditions? (ii) How does their molecular machinery adapt to changing environmental conditions such as temperature, pressure, and radiation? and (iii) Could a terrestrial microorganism colonize Mars?

## 2. Materials and methods

### 2.1. Sample collection

**Figure 1:**
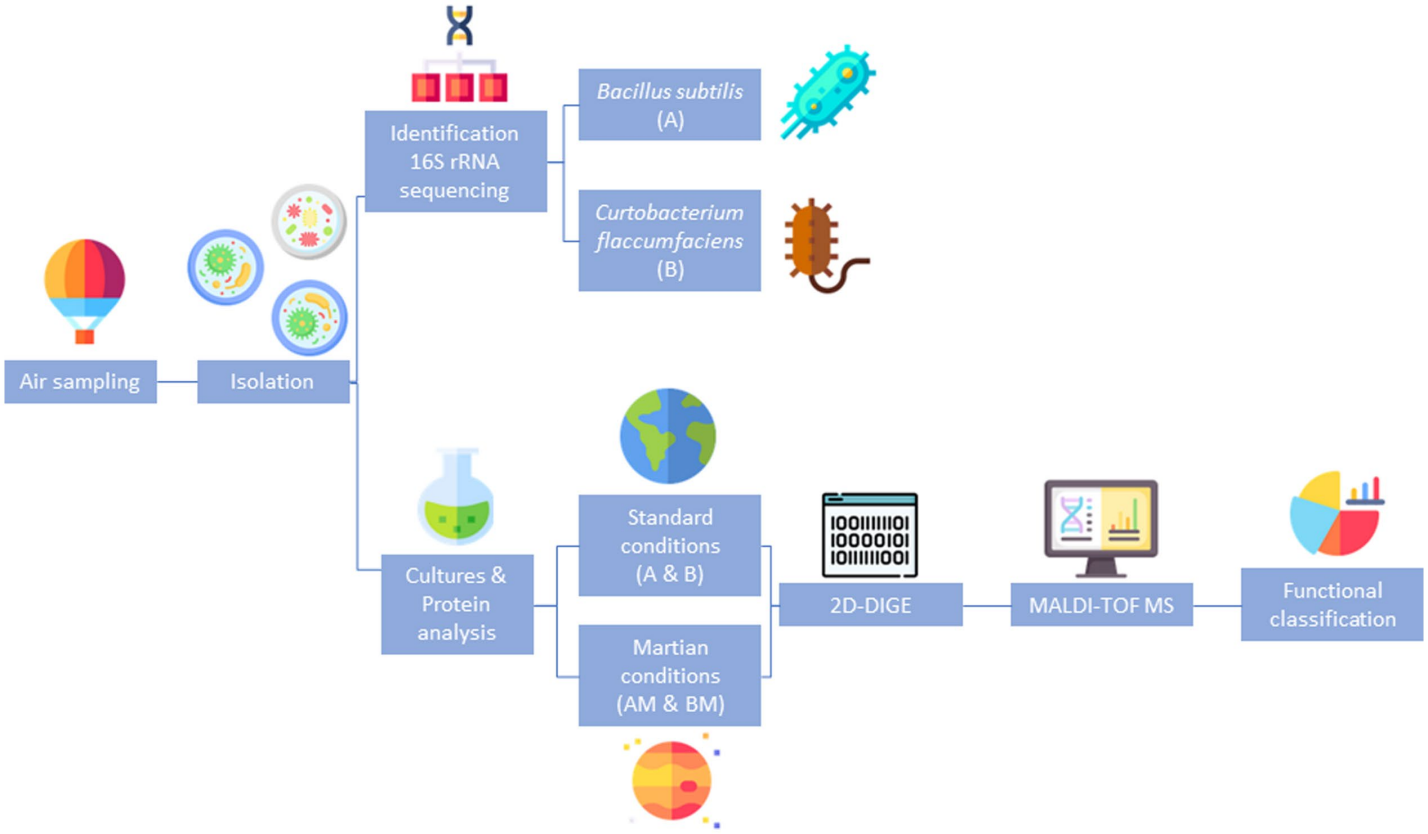
Summary of the overall experimental strategy. Schematic representation of the experimental design followed to identify differentially expressed proteins in the response of *Bacillus* and *Curtobacterium* to exposure to Martian conditions.

A summary of the overall experimental strategy is represented in Figure 1.

The stratospheric air samples were taken on August 24, 2020 at the Villaverde aerodrome, (Toledo, Spain), using a stratospheric balloon as part of the CAMELIA-MICRO project (PID2019-104205GB-C22) as described elsewhere (González-Toril et al., 2020). The Earth stratosphere has conditions somewhat similar to those of Mars in terms of temperature, pressure, and UV radiation, and therefore microorganisms capable of surviving in it could potentially grow on Mars (Wainwright et al., 2003; Griffin, 2004; Khodadad et al., 2017). Taking samples from the stratosphere and simulating Martian environments are difficult tasks to be carried out, but the development of stratospheric balloons (Smith and Sowa, 2020) and the construction of sampling chambers (Bryan et al., 2014; Della Corte et al., 2014) significantly facilitate this work. The air outside of the stratospheric balloon was collected using a sampling chamber incorporated into the balloon gondola. The sampling chamber was coupled to a vacuum pump that was activated when the balloon reached the desired height (between 35,613 m and 16,333 m for 1 h). Aerosols were collected on three Petri dishes with R2A culture medium (composition (g/L): 0.5 g of yeast extract/0.5 g of peptone/0.5 g of casamino acids/0.5 g of glucose/0.5 g of soluble starch/0.5 g of sodium pyruvate/0.5 g of KH_2_PO_4_/0.05 g of MgSO_4_·7H_2_O) (Garcia-Descalzo et al., 2012) and incubated at 30°C. Thus, a small sample of the microorganisms present in this layer of the atmosphere could be obtained. Several bacterial colonies could be observed in Petri dishes, of which two were isolated and designated A and B. These bacteria were frozen with 40% glycerol and used for the following experiments. All procedures were performed using ethanol-sterilized tools and sterilized gloves in a UV-irradiated laminar flow hood. To control for laboratory contamination, 1 liter of MilliQ rinse water was subjected to identical analytical procedures.

### 2.2. DNA extraction and sequencing

Bacterial DNA was extracted and purified with a FastDNA SPIN Kit for Soil (MO BIO Laboratory, Inc.). DNA concentration was determined using a NanoDrop 2000p (Termofischer). Amplifications of DNA were performed using standard PCR methods as in Angulo-Preckler et al. (2020). Primers used to amplify the prokaryotic 16S rDNA were 27F (AGAGTTTGATCMTGGC) and 1492R (TACCTTGTTACGACGACTT) (Lane, 1991).

16S rDNA PCR amplicons were cloned using TOPO TA Cloning Kit (Invitrogen, Carlsbad, CA), and plasmid DNA was bidirectionally sequenced with a 48 capillary sequencer ABI 3730 XL (Applied Biosystems). Read-lengths of up to approximately 1,000 bp were achieved. Sequences were analyzed with UCHIME, to identify and remove chimeric reads, and classified to eliminate those that could be considered contaminants (Edgar et al., 2011).

Sequences were analyzed using BLAST at the NCBI database.^1^ Representative sequences were aligned using the software of Clustal Omega (Sievers et al., 2020), the results were corrected manually and alignment uncertainties were omitted in the phylogenetic analysis. Their phylogenetic relationship was analyzed using the software MEGA 11 (Tamura et al., 2021) with parsimony, neighbor-joining, and maximum likelihood analyses. In all cases, general tree topology and clusters were stable, and reliability of the tree topologies was confirmed by bootstrap analysis using 1,000 replicate alignments. A consensus tree was generated. The isolated species were determined to be: A-*Bacillus subtilis* and B-*Curtobacterium flaccumfaciens*.

### 2.3. Bacterial growth experiments

The isolated A and B colonies were harvested from plates by gently scraping the colonies out using a cotton stick and cultured in glass flasks with 50 mL of TB culture medium (composition (g/L): yeast extract 24.0 g; tryptone 12.0 g; sodium pyruvate 4.0 g; K_2_HPO_4_ 9.4 g; KH_2_PO_4_ 2.2 g. Add 4 mL glycerol). The flasks were incubated at 30°C with continuous agitation at 120 rpm. The O.D. at 600 nm was checked until cultures reached the stationary growth phase. Growth curves are shown in Supplementary Figure S1. Four replicates of each culture were prepared.

### 2.4. Tolerance assays in MARTE chamber

**Figure 2:**
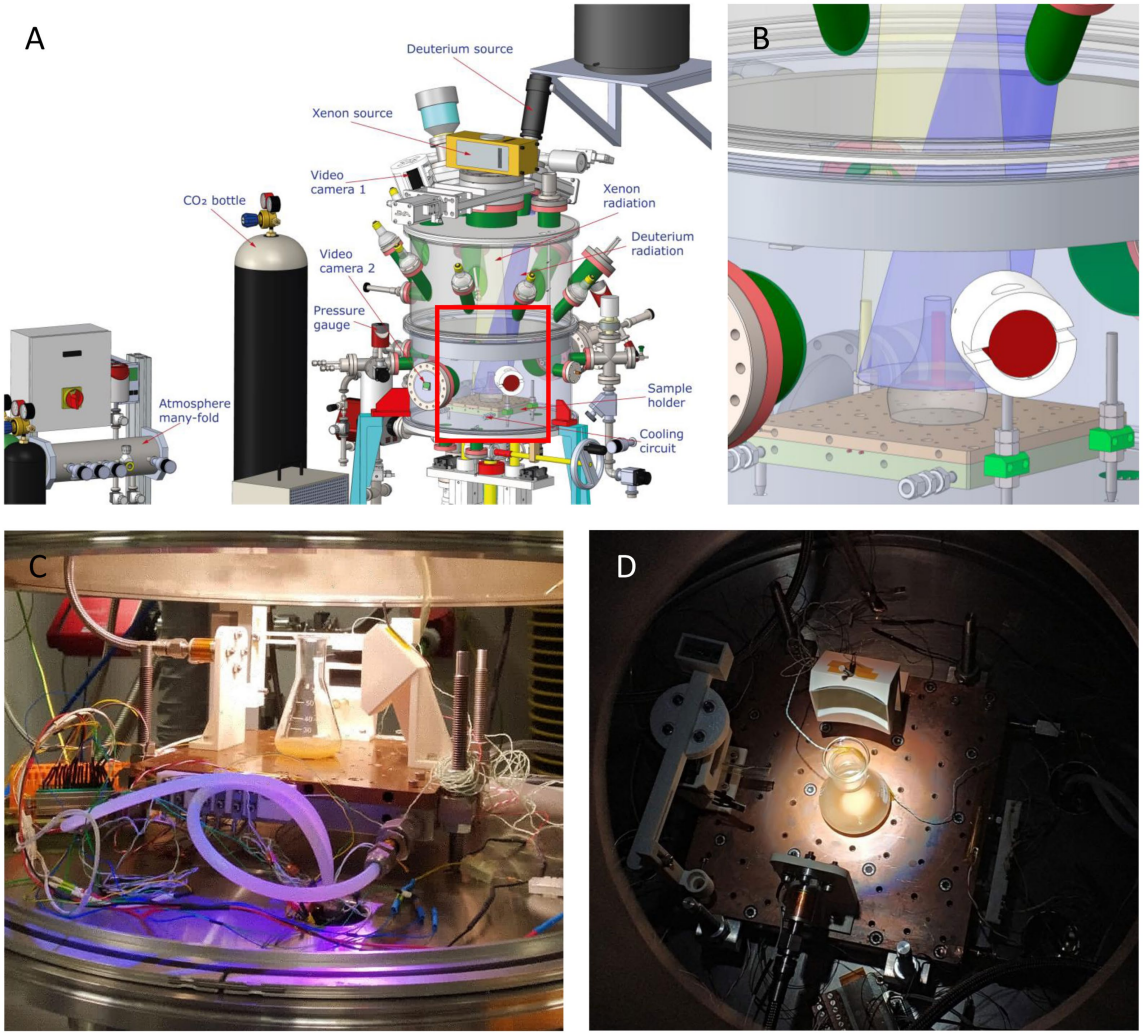
Tolerance Assays in MARTE chamber. **(A)** Schematic drawing of the MARTE chamber, indicating the positions of the deuterium and xenon sources and the direction of the radiation they emit. The position of the equipment that maintains the CO2 atmosphere, the pressure gauge, the inlet and outlet of the cooling circuit and the sample holder are also showed. **(B)** Detail of the inset in Figure 2A representing the sample holder where the bacterial culture is placed. **(C)** Image of the culture of sample A in the MARTE chamber taken with the video camera 2. **(D)** Image of the culture of sample B in the MARTE chamber taken with the video camera 1.

To determine the effects of the Martian environment on bacteria, cultures grown in pressure, vacuum and temperature resistant glass flasks (SCHOTT-DURAN^®^) with 50 mL of TB culture medium were introduced in the MARTE chamber (Figure 2). The main differences between the conditions used in the chamber MARTE and the conditions reported on the planet Mars are summarized in Table 1. The chamber parameters were: Temperature: −15°C; Pressure: 8 mbar; CO_2_ atmosphere: >95%; Radiation: Xenon lamp (Hamamatsu model 150w L11033 lamp (185–2000) nm: 666 W/m^2^, UVA (320–395 nm): 1.46 W/m^2^, UVB (265–322 nm): 0.33 W/m^2^, UVC (225–280 nm): 0.25 W/m^2^). Deuterium lamp (Hamamatsu model 140 W L10366 lamp (115–400) nm maximum 160 nm (95% emission) UVA + UVB + VIS (305–450 nm): 5.11 W/m^2^). Time: Radiation 14 h, without radiation 8 h (nighttime), radiation 2 h (Sobrado et al., 2014; Sobrado, 2020). The light source is placed on top of the MARTE chamber. Several DN40CF flanges are placed on the body of the upper ring, pointing towards the sample to simulate the position of the sun with respect to Mars. During irradiation, cell suspensions were mixed continuously to avoid shading effects. As the culture medium contains glycerol and salts it does not freeze and this prevents the cells from lysis. These new samples were named AM (sample A subjected to Martian conditions) and BM (sample B subjected to Martian conditions) (Figure 2). Four experiments (independent cultures) were prepared for each bacterial species.

**Table 1:** Main differences between the conditions used in the chamber MARTE and the conditions reported on the planet Mars.

	MARTE chamber	Planet Mars	References
Temperature (°C)	−15	(10 in summer) −(−100 in winter at the poles)	Forget et al. (1995); Aerts et al. (2014)
Pressure (mbar)	8	7–20	Sobrado (2020)
Atmosphere	100% CO_2_	95% CO_2_; 2.7% N_2_; 1.6% Ar; 0.13% H_2_0; 0.08% CO	Sobrado (2020)
Essential elements	C, H, N, O, P, S	C, H, N, O, P, S	McGlynn et al. (2012)
Solvent	TB culture medium	Brines	Martin-Torres et al. (2015)
Source of energy	Xenon lamp 666 W/m^2^	Solar radiation 550 W/m^2^	Cockell et al. (2016)

### 2.5. Determination of microbial survival by standard plate counting

The survival of microorganisms was determined by standard plate counting as in Cortesão et al. (2021) (Supplementary Figure S2). Serial dilutions (1,10) were seeded on Petri dishes with R2A culture medium and incubated for 2 days at 30°C. Colony forming units (CFU) were counted, and the colony forming units per mL (CFU/mL) were calculated. The survival fraction was calculated as XM/X, in which XM is the CFU/mL of *B. subtilis* (AM) or *C. flacumfaciens* (BM) in Martian conditions and X is the CFU/mL of *Bacillus subtilis* (A) or *C. flacumfaciens* (B) in standard conditions. Four replicates of each culture were prepared. Statistical analyses were performed using GraphPad Prism version 7.0 (GraphPad Software, La Jolla California United States, www.graphpad.com).

### 2.6. Extraction of the soluble protein fraction

The preparation of cell extracts and protein determination was performed as described in Cid et al. (2010). The pellets of each flask were obtained by centrifugation for 20 min (10,000 × *g*; 4°C), washed and lysed in buffer A (20mMTris-HCl, pH 7.6; 140 mM potassium chloride; 2 mM benzamidine; 1 mM EDTA; 10 μg/mL pepstatin A, leupeptin, and antipain), using a French Press at 4°C. Cell debris was removed by centrifugation at 11,000 × *g* for 10 min to obtain a supernatant, and the protein extracts were processed using a 2D Clean-Up kit (GE Healthcare, Spain). The pellets were frozen and stored at −80°C. Protein concentration was quantified using the Qubit fluorometer (Invitrogen) previously calibrated according to the manufacturer’s protocol. The samples were adjusted to a protein concentration of 5–10 mg/mL. All steps were carried out at 4°C.

### 2.7. 2D-DIGE

Proteins from bacterial cells were analyzed by 2D-DIGE experiments, to compare cultures to those cultures subjected to Martian conditions. Four biological replicates were obtained from each different culture. Samples (40 μg) from each experimental condition were made to 7 M urea/2 M thiourea, centrifuged, and labeled with Cy5 or Cy3 CyDye DIGE Fluor minimal dyes (Cytiva, United States); according to the Ettan DIGE protocol. A pooled standard of samples A + AM or B + BM was labeled with Cy2 (Supplementary Figure S3). After DTT addition, pairs of experimental and control samples were mixed and subjected to 2DE combining horizontal slab gel isoelectric focusing with SDS-PAGE as previously described (García-Descalzo et al., 2022).

Samples were applied to pH gradient strips for IEF. Carrier ampholyte urea IEF was performed using immobilized pH 4–7 gradient strips. After the first dimension, the IEF strips were equilibrated for the second dimension in two successive SDS equilibration buffer solutions containing Tris buffer 100 mM, urea 6 M, and glycerol 30% (w/v). The second dimension in SDS-PAGE was carried out on 12% acrylamide (2.6% crosslinking) gels (1.0 mm thick). Gels were stained with Coomassie Blue reagent for protein identification.

### 2.8. Protein identification

Protein gel images were processed and analyzed using the DeCyder Differential In-gel Analysis (DIA). The spots were co-detected, quantified, normalized, and matched between the four replicate experiments. Protein abundance changes between samples from each strain (A/AM ratio) or (B/BM ratio) were examined by ANOVA test. Spots present in at least three of four gels per group, with significant ANOVA test (*p* ≤ 0.01), and an averaged ratio ± 2 were considered and selected for further MS analysis. Those spots selected were excised, digested with trypsin, and identified by matrix-assisted laser desorption/ionization time-of-flight mass spectrometry (MALDI-TOF MS). Combined peptide mass fingerprint and MS/MS ion search modes were applied against the NCBInr databases. The MASCOT (Matrix Science, London, United Kingdom) database search algorithm was used for protein identification [search parameters: enzyme: trypsin; fixed modifications: carbamidomethyl (C); variable modifications: oxidation (M); mass values: monoisotopic; protein mass: unrestricted; peptide mass tolerance: 80 ppm]. Protein scores higher than 66 were significant (*p* < 0.05) in the Mascot database search algorithm (Matrix science, UK) (Supplementary Table S1).

## 3. Results

### 3.1. Microbial survival to Martian conditions

The microbial survival after exposure of cultures to Martian conditions was represented in Supplementary Figure S2. Both bacterial species survived exposure to Martian conditions for 24 h. *Bacillus subtilis* was quite resistant to Martian conditions, although both cultures A and AM presented significant differences (*p* < 0.0001; *t* = 337.1 df = 6) in the number of CFU observed. The species *Curtobacterium flacumfaciens* was much less resistant than *Bacillus subtilis* and the differences in the number of CFU between B and BM were significant (*p* < 0.0001; *t* = 29.84 df = 6). Statistical differences were studied by student’s *t* test. The average of four samples (*n* = 4) was reported in Supplementary Figures S2A,B.

### 3.2. Molecular machinery of *Bacillus subtilis* and *Curtobacterium flacumfaciens* under standard and Martian conditions

To assess whether these bacterial species were capable of adapting in the environmental conditions of the planet Mars, proteomes from cultures under standard conditions (samples A and B) and cultures submitted to the Martian environment simulation chamber (samples AM and BM) were compared (samples A vs. AM, and samples B vs. BM) (Figure 2). To establish this comparison, both the expression levels of each protein, and the variation in their distribution within the different functional categories were considered. The functional classification of proteins has been done following the nomenclature of protein-coding genes (Kunst et al., 1997).

**Figure 3:**
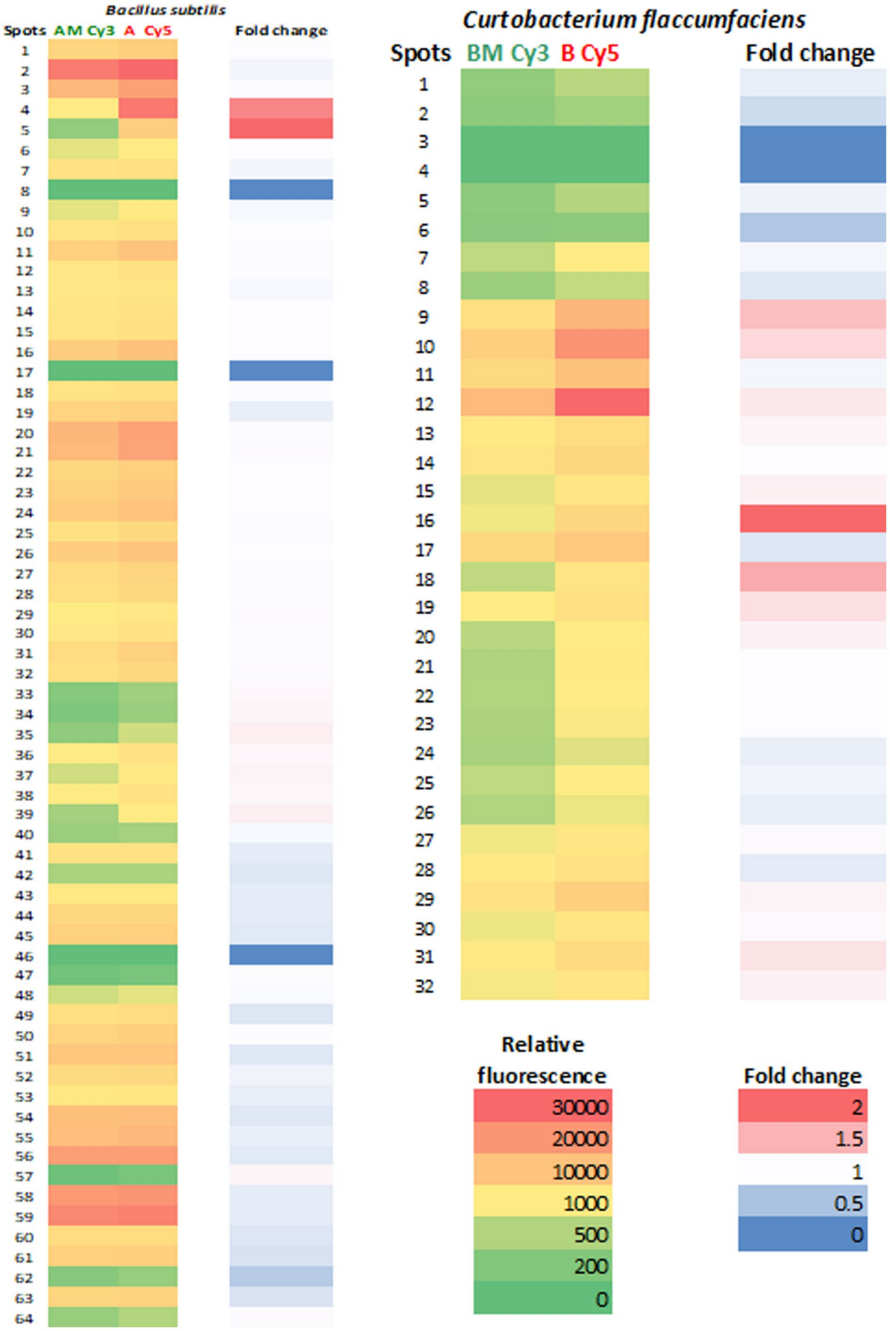
Fluorescence intensity of spots in 2D-DIGE gels. A heat map of ratios from 2D-DIGE experiments of the proteins over-expressed at standard conditions and Martian conditions. The relative fluorescence of each spot was represented in red/green color. The fold change (red/blue) is the value of standard/Martian ratio obtained in the 2D-DIGE experiments so that values closer to 2 indicate higher protein expression.

It is noteworthy that, in both bacterial species, protein expression levels were lower in extracts obtained from cells subjected to Mars conditions than in extracts from standard cultures (Figure 3). Some proteins did not change their expression levels, for example, spots 8 and 17 of *Bacillus* and 3 and 4 of *Curtobacterium*. The greatest differences in expression levels were observed in spots 4 (GroEL) and 5 (Dihydrolipoyl dehydrogenase) of *Bacillus* and spots 16 (Peroxiredoxin) and 18 (Envelope stress response protein) of *Curtobacterium* (Supplementary Table S1).

### 3.3. Proteome-inferred microbial activity

An important general observation is that when comparing the proteomes, the *Bacillus* set of proteins contained a greater number of proteins and with more diverse functions than the *Curtobacterium* proteome (Supplementary Figure S3). This particularity demonstrates that *Bacillus* contains a large repertoire of molecules that exert different functions and gives this bacterium greater molecular versatility.

**Figure 4:**
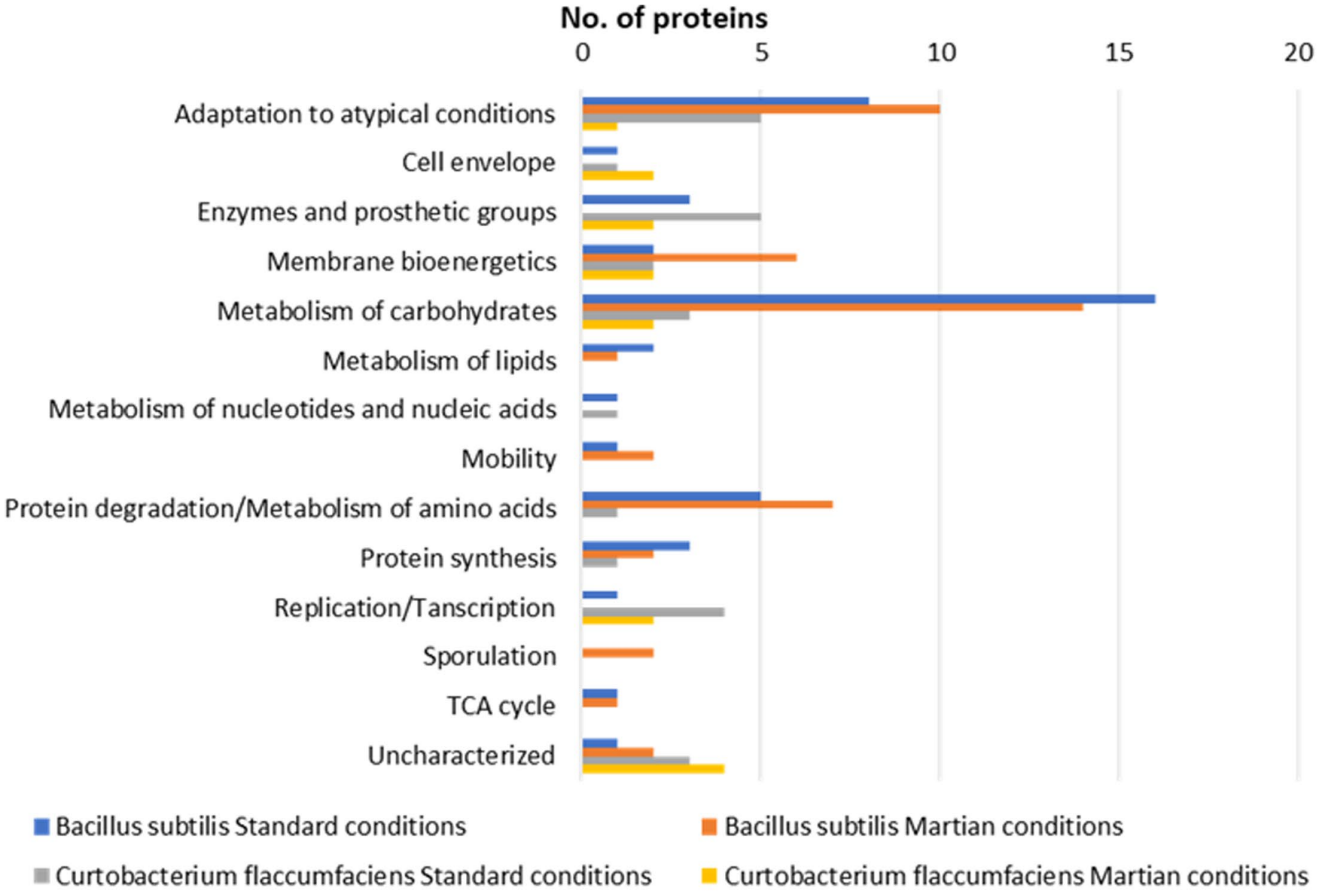
Functional classification. Representation of the number of proteins identified in 2D-DIGE gels from cultures of *Bacillus subtilis* and *Curtobacterium flacumfaciens* under standard and Martian conditions.

The identified proteins in these bacterial species could be classified within the next different functional categories (Figure 4; Supplementary Table S1).

#### 3.3.1. Adaptation to atypical conditions

The proteins of this group are related to the stress response that can induce the death of the bacteria. Proteins of this category appeared in all proteomes, although they were the majority in the proteomes obtained from cultures under Martian conditions. The proteolytic subunit of the ATP-dependent endopeptidase Clp appeared in the proteomes of both bacteria (*Bacillus* and *Curtobacterium*). This peptidase degrades misfolded proteins, acting similarly to chymotrypsin (Zellmeier et al., 2006). In addition, general proteins secreted in response to stress, and proteins of the GrpE family were also identified under Martian conditions. These proteins participate together with DnaK in the response to hyperosmosis and heat shock; they are chaperones or Heat shock proteins that prevent the aggregation of proteins denatured by stress (García-Descalzo et al., 2014). In these bacteria, the presence of these proteins protects cells from the stress suffered by constant radiation and low temperatures.

This category also includes proteins responsible for eliminating or degrading certain molecules that can be harmful to the cell. The main enzyme in this group is superoxide dismutase, which catalyzes the degradation of superoxide to obtain oxygen and hydrogen peroxide. This enzyme is part of a universal antioxidant mechanism required for life-forms to survive the hyperoxic conditions of desiccation in Earth’s atmosphere (Horne et al., 2022).

It was also noteworthy the presence of the enzyme thiol peroxidase in *Bacillus*, which is oxidized by hydrogen peroxide, reducing this molecule and preventing the oxidative stress in the cell. It is also related to the activation of certain genes (Ruiz-Blas et al., 2023). The presence of these proteins was very similar in both conditions, although, as in the previous cases, they were overexpressed in standard conditions (Figure 4).

#### 3.3.2. Cell envelope

Proteins in this category are related to the formation of the peptidoglycan bacterial wall that protects the bacteria from external agents. Both microorganisms *Bacillus* and *Curtobacterium* are gram-positive bacteria which means that their peptidoglycan layer is very thick. A protein representative of this category in the *Bacillus* proteome was the enzyme D-alanine ligase, which joins two molecules of alanine through ATP hydrolysis. This reaction is fundamental in the biosynthesis of peptidoglycans, which are later used to form the cell wall (Yong-Fang et al., 2007).

#### 3.3.3. Enzymes and prosthetic groups

The proteins contained in this category are related to the metabolism and synthesis of coenzymes and prosthetic groups. One of the proteins that appeared in *Bacillus* was the enzyme (R, R)-butanediol dehydrogenase, which utilizes NAD^+^ and (R, R)-butane-2,3-diol as substrates, to obtain NADH, H^+^, and (R)-acetoin as products (Höhn-Bentz and Radler, 1978). Several proteins of this group were also identified in *Curtobacterium*. For example, some enzymes with transferase activity such as PEP-utilizing enzyme (Anderson, 2005), and others such as methylenetetrahydrofolate reductase, metallo-hydrolase and L-lactate dehydrogenase (Chen et al., 2021).

#### 3.3.4. Membrane bioenergetics

This group of proteins is involved in the processes of the electron transport chain and ATP synthesis. Their function is the establishment of an electrochemical gradient that is used to synthesize ATP that will later be used in cellular processes as an energy source. The final acceptor of the electron transport chain under standard conditions is oxygen. But under Martian conditions, the atmosphere is mainly composed of CO_2_, and it has been reported that *Bacillus subtilis* can survive in anaerobic conditions using nitrate as the final electron acceptor (Cruz Ramos et al., 2000). This fact could be demonstrated in these experiments by the presence of NADPH-dependent oxidoreductase enzymes, as NADPH is the preferred cofactor in anabolic conditions (Sellés Vidal et al., 2018). It could be concluded that in the simulation chamber for Martian conditions the bacteria were not carrying out the processes of cellular respiration, but fermentation. The observation of this group of proteinases in both bacteria implies that they are active and producing energy, either under aerobic or anaerobic conditions.

#### 3.3.5. Metabolism of carbohydrates

The proteins included in this category are related to the breakdown of carbohydrates to obtain energy in the form of ATP. Therefore, the presence of these proteins indicates that the cell is functioning optimally since it is producing energy to carry out its basic functions. It can be observed that this is the majority group both under standard and Martian conditions, although the percentage of proteins is slightly lower in the latter situation. Within this group, the most numerous proteins are those involved in glycolysis, such as fructose-1,6-bisphosphate, present in both proteomes (Fujita and Freese, 1979). In the 2D-DIGE experiments, it was observed that the expression of these proteins was higher in the extracts of standard conditions in both proteomes.

#### 3.3.6. Metabolism of lipids

The beta-oxidation of fatty acids is the metabolic pathway that breaks down complex fatty acids in the presence of oxygen to generate energy in the form of ATP. In addition, these reactions break down fatty acids to Acetyl-CoA, which is a substrate for some enzymes involved in the Krebs cycle.

One of the enzymes isolated in the proteome obtained from cultures under standard conditions that participates in this process was the enoyl-CoA hydratase, which transforms a double bond into a single one and a hydroxyl radical by adding a water molecule (Frandi et al., 2011).

Regarding the proteome obtained in Martian conditions, the phosphate acetyltransferase, which transforms acetate into acetyl-CoA, was detected and also appears in standard conditions. This reaction does not need to take place under aerobic conditions, and then acetyl-CoA can be used as a starting molecule in fermentation (Madigan et al., 2014).

This group is more present under standard conditions than under Martian conditions, and, again, the proteins that appear in both proteomes are more expressed in bacteria grown under standard conditions according to 2-DIGE electrophoresis.

#### 3.3.7. Metabolism of nucleotides and nucleic acids

In this category, only one protein from *Bacillus*, the adenylate kinase, was isolated, both in the proteome from the culture under standard conditions, and in the proteome obtained from the culture under Martian conditions. This enzyme catalyzes the transfer of a phosphate group from ATP to AMP to obtain two equivalents of ADP. This reaction regulates cellular ATP homeostasis, thus making the cell produce energy to carry out all cellular processes (Davlieva and Shamoo, 2009).

In the *Curtobacterium* proteome, the ATP-binding cassette domain-containing protein was identified. This ubiquitous group of membrane proteins takes part in diverse biological functions. Their main role involves the unidirectional translocation of compounds across cellular membranes in an ATP-coupled process (Lewis et al., 2012).

#### 3.3.8. Protein degradation/metabolism of amino acids

These proteins perform functions related to the degradation of proteins and amino acids to later synthesize new proteins. Amino acids are broken down because they are not needed or because the cell needs energy and cannot get it from the main pathways of carbohydrate metabolism.

**Figure 5:**
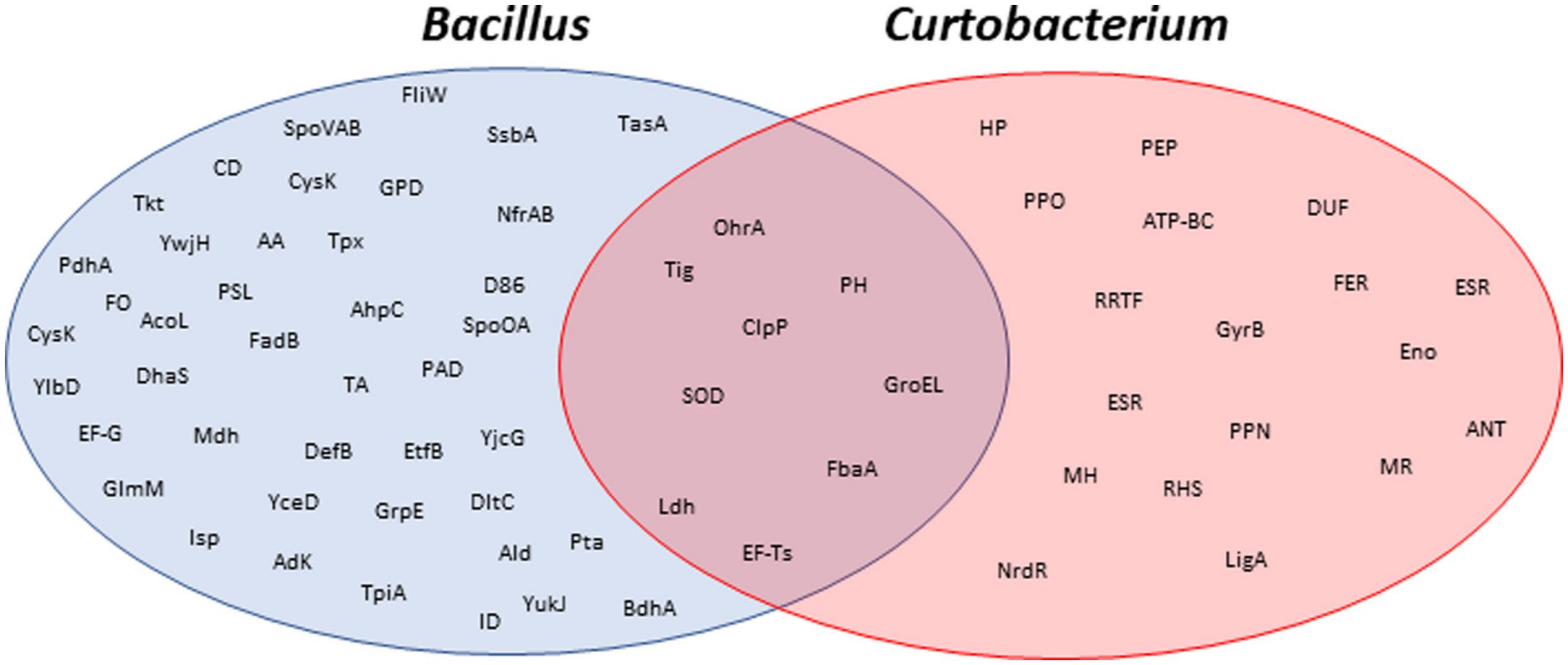
Venn diagrams of the identified proteins. Proteins identified by MALDI-TOF MS in 2D gels from *Bacillus subtilis* and *Curtobacterium flacumfaciens.* Protein abbreviations are detailed in Supplementary Table S1.

This was the second largest group of proteins identified in both conditions, implying that proteins and amino acids are being broken down to synthesize other proteins that the cell needs or as an alternative method of obtaining energy. It can then be confirmed that under Martian conditions bacteria continue to carry out these processes in a similar way as they do under standard conditions since they are common to both proteomes (Figure 5). One of those proteins that appeared in both proteomes was alanine dehydrogenase. This enzyme catalyzes the reversible oxidative deamination of L-alanine to pyruvate. It is a key factor in the assimilation of L-alanine as an energy source through the tricarboxylic acid cycle during sporulation (Uhlenbusch et al., 1991; Borriss et al., 2018). In these cases, comparing the fluorescence intensity of the 2D-DIGE spots, it could be observed that the expression of these proteins was always higher under standard conditions (Figure 5). The investigation of radiation-induced damages of proteins has a long tradition in radiation biology, especially enzymes containing SH or SS groups (Durchschlag et al., 1996). This may be one of the fundamental causes of the decrease in fluorescence observed in protein gel spots obtained with cultures subjected to Martian conditions.

#### 3.3.9. Protein synthesis

The presence of these molecules implies that bacteria are synthesizing proteins to carry out different cellular processes. Proteins such as the elongation factors G, Ts, and Tu were isolated in the proteomes of *Bacillus* and *Curtobacterium* under standard conditions (Doherty et al., 2006). Also in this category are the proteins that are involved in the synthesis of amino acids, since they are the basis for the formation of proteins, such as cysteine synthase, present in both *Bacillus* proteomes (Tanous et al., 2008).

This group was more present under standard conditions than under Martian conditions and, once again the proteins that appeared in both proteomes were more expressed in bacteria grown under standard conditions according to 2-DIGE electrophoresis.

#### 3.3.10. Replication/transcription

These proteins are involved in processes related to the transmission of genetic information; therefore, their presence indicates that there is a reproduction of the bacterium if DNA replication is taking place, or that the transcription of DNA to RNA is taking place so that proteins can be synthesized. Two proteins belonging to this group were isolated in *Bacillus* under Martian conditions. One of them was the response-regulating protein aspartate phosphatase A, which intervenes at the transcription level. This phosphatase inhibits the protein Spo0A which is responsible for spore formation (Howell et al., 2003). The other identified protein was the single-stranded DNA-binding protein B, which is necessary for the optimal *Bacillus subtilis* chromosome replication since it acts as an extra factor for the exchange of homologous DNA strands (Yadav et al., 2014).

No proteins from this group were identified in *Curtobacterium*, which may mean that genetic transmission is inhibited in this bacterium, or that the proteins of this group could not be detected.

#### 3.3.11. TCA cycle

The proteins classified in this category are those that participate in the Krebs cycle or cycle of tricarboxylic acids. It is a metabolic pathway that cell use to obtain energy in the form of GTP under aerobic conditions from carbohydrates, lipids, and proteins. Finding these enzymes in the proteome of bacteria grown in the MARTE chamber was not expected, since the conditions were anaerobic. An enzyme that appeared in the proteome of bacteria grown under standard conditions was the NADP^+^-dependent enzyme isocitrate dehydrogenase, which oxidizes and decarboxylates isocitrate in one of the stages of the Krebs cycle (Bartholomae et al., 2014). In addition, other types of enzymes that degrade carbohydrates, lipids, and proteins to molecules that serve as a substrate for enzymes involved in the Krebs cycle were identified in *Bacillus*. Once more, no proteins in this category were found in the *Curtobacterium* gels.

#### 3.3.12. Mobility

*Bacillus subtilis* is a species capable of forming a flagellum that serves to move through the culture medium. In this case, only one protein related to this process has been detected, and it has been isolated in the proteome obtained from the culture under Martian conditions. The protein is flagellin, which is the subunit that polymerizes to give rise to filaments that form the bacterial flagellum. The hag gene is the one that encodes this protein in *Bacillus subtilis* and although in this case it only appears in extreme conditions, its expression does not have to be linked to this type of situation (Titz et al., 2006).

No proteins from this group were detected in *Curtobacterium*. Although *Curtobacterium* cells are small irregular rods with lateral flagella (Yong-Fang et al., 2007), their mobility is probably less agile than that of *Bacillus*.

#### 3.3.13. Sporulation

Proteins belonging to this category have functions related to spore formation. The spores serve the bacteria to reduce their functions and their biological activity when the conditions for living are not optimal, remaining in a latent state in which they only conserve the genetic material, but do not carry out vital functions (Madigan et al., 2014). Proteins belonging to this group have only been detected in the proteome obtained from the culture in Martian conditions. As the conditions to which the bacteria were subjected were very extreme, some of them may have transformed into spores to preserve the genetic material and resist these conditions. Despite this, it is a minority group within the proteome, since it only accounts for 5% of the proteins obtained in total. The proteins obtained favor sporulation, such as those of the YlaJ family or the spoVABEA sporulation proteins (Johnson et al., 2017). This group also includes the proteins involved in biofilm formation, such as the protein that synthesizes the main components of the biofilm matrix. They form fibers that bind cells together and create a protective layer on the surface of the culture that protects the rest of bacteria. Both for the formation of this film and the spores, bacteria usually harmonize thanks to the mechanism of “quorum sensing”, an extracellular communication process in which cells excrete substances known as auto-inductors that serve surrounding cells as a chemical signal to induce collective gene expression. This molecular communication has generated a keen interest in the scientific community over recent years (Kalamara et al., 2018; Ryan-Payseur and Freitag, 2018; Mukherjee and Bassler, 2019; Špacapan et al., 2020).

*Curtobacterium flacumfaciens* is a non-spore-forming microorganism (Khanal et al., 2023). Therefore, no proteins of this category were found in the proteome of this bacterium.

#### 3.3.14. Uncharacterized

This category includes both indeterminate proteins, such as hypothetical proteins, as well as proteins that, although their position in the genome is known and characterized, do not have a specific function, such as those of the Yukj or YlbD families (Ijaq et al., 2015).

## 4. Discussion

In recent years it has been determined that vegetative cells of bacteria (*Deinococcus radiodurans*, *Escherichia coli*), and endospores of several species of *Bacillus* and budding yeast (*Saccharomyces cerevisiae*) are capable of surviving in a simulator of Martian conditions (Horne et al., 2022). This study complements this discovery since it not only reproduces the test of subjecting the bacterial culture to conditions of radiation, temperature, and atmosphere similar to those of the planet Mars, but also studies how the cellular machinery of microorganisms that could populate the Martian cryosphere changes. Our study has been restricted to the first 24 h because after the first hours of exposure, resistance elements such as spores begin to develop, and the cellular machinery would be different from that of vegetative cells. Sporulation occurs after different stress signals, which requires previous bacterial growth. The time needed to obtain resistant spores has been reported between 15 h (at 40°C) and 40 h (at 27°C), depending on the temperature (Gauvry et al., 2019). In addition, it is known that the structure, composition and properties of the spores are determined by the environmental conditions in which spores are formed (Bressuire-Isoard et al., 2018).

This research compares the proteomes obtained from the culture grown under standard conditions and the culture grown under Martian conditions considering both the protein expression levels and the variation in their distribution within the different functional categories. The experiments reported here were carried out with a model bacterial species, *Bacillus subtilis*, whose molecular machinery is well known. In addition, these experiments were carried out with another more unknown bacterium, such as *Curtobacterium*. This bacterial species had never been subjected to this type of research before.

From the results obtained, it can be concluded that both *Bacillus subtilis* and *Curtobacterium flacumfaciens* would be capable of surviving on Martian cryosphere conditions with a moderate level of stress for at least the time that our test lasted (24 h). This observation opened new questions. What strategy did each of the two species follow to cope with the Martian cryosphere conditions?

From the results obtained, it can be concluded that both species share common mechanisms of resistance (Figure 5). For example, the two bacteria synthesized a battery of proteins for their adaptation to atypical conditions. Among them, it is worth noting those that have an antioxidant function such as superoxide dismutase. Under Martian conditions, the proteins that allow metabolism to be maintained under anaerobic conditions are remarkable.

### 4.1. *Bacillus* under Martian conditions

The study of the isolated proteins determined that this species continued carrying out the bacterial processes that it presented in standard conditions after entering the Martian conditions simulator, although some proteins expressed as a defense mechanism also appeared. *Bacillus subtilis* submitted to Martian conditions increased the number of proteins belonging to the categories of Adaptation to atypical conditions, Membrane bioenergetics, and Protein degradation (Figure 4). The presence of proteins related to Motility and Sporulation were also significant which means that cells tended to move, or develop resistance forms such as spores.

### 4.2. *Curtobacterium* under Martian conditions

However, the bacterial species *Curtobacterium flacumfaciens* developed different strategies from those of *Bacillus*. The number of proteins in the cell envelope increased, trying to protect cells from the extracellular environment. It was also noteworthy the large number of unknown proteins detected (Figure 4). Despite being well known as a plant pathogen, the molecular machinery of *Curtobacterium* is largely unknown compared to that of *Bacillus*. It is necessary to investigate its genome and proteome to conclude the resistance strategies of this microorganism.

## 5. Conclusion

– The analyzed bacterial species (*Bacillus subtilis* and *Curtobacterium flacumfaciens*) withstood the environmental conditions of Martian cryosphere, i.e., subglacial lakes.
– The *Bacillus* proteome contained a larger number of proteins with more diverse functions than the *Curtobacterium* proteome.
– Proteins from bacteria under standard conditions were overexpressed relative to proteins from cells under Martian conditions. Their protein synthesis systems must be impaired in Martian conditions.
– Both species share common resistance mechanisms such as protein synthesis for the adaptation to atypical conditions, or fermentative metabolism under anaerobic conditions.
– The resistance strategy of *Bacillus* consists of increasing its stress response, membrane bioenergetics, biomolecule degradation, increasing its mobility and finally the formation of resistance biofilms or spores.
– In contrast, the *Curtobacterium* approach consists of strengthening the cell envelope, trying to protect the cells from the extracellular environment.
– These findings are relevant due to their application in planetary protection. They warn about the possible risk of contamination by microorganisms in the missions that are being carried out for the exploration of planets such as Mars. All these missions should ensure that scientific investigations related to the origin, evolution, and distribution of life are not compromised.

## Data availability statement

The mass spectrometry proteomics data are deposited to the ProteomeXchange Consortium via the PRIDE repository with the dataset identifiers PXD027245 (samples A and AM) and PXD027249 (samples B and BM).

## Author contributions

CC conceived and planned the experiments and managed the acquisition of funds. FR-B, VM-H, and EG-L performed the bacterial cultures, genomics experiments and proteomics gels. JS built the chamber MARTE and controlled it during the experiments. EM-A and AA carried out MALDI-TOF experiments. CC and FR-B wrote, revised and edited the manuscript. All authors contributed to the article and approved the submitted version.

## Funding

This research has been funded by the Spanish State Research Agency (AEI) project nos. PID2019-104205GB-C22, PID2020-114047GB-100, and MDM-2017-0737 CAB (CSIC-INTA), Unidad de Excelencia María de Maeztu. EG-L is recipient of a Fellowship (PTA2016-12325-I). This work is part of the CSIC Interdisciplinary Thematic Platform (PTI) Polar zone Observatory (PTI-POLARCSIC) activities.

## Conflict of interest

The authors declare that the research was conducted in the absence of any commercial or financial relationships that could be construed as a potential conflict of interest.

## Publisher’s note

All claims expressed in this article are solely those of the authors and do not necessarily represent those of their affiliated organizations, or those of the publisher, the editors and the reviewers. Any product that may be evaluated in this article, or claim that may be made by its manufacturer, is not guaranteed or endorsed by the publisher.

## Footnotes

^1^ http://ncbi.nlm.nih.gov/BLAST

## Abbreviations

2D-DIGE: two-dimensional fluorescence difference gel electrophoresis
MALDI-TOF MS: matrix-assisted laser desorption/ionization-time of flight (MALDI-TOF) mass spectrometry (MS) Protein abbreviations are detailed in Supplementary Table S1.
