# Supplementary material for: Bacterial molecular machinery in the Martian cryosphere conditions
Source: Front Microbiol. 2023 Jul 26;14:1176582. doi: 10.3389/fmicb.2023.1176582 (PMC10569478; doi:10.3389/fmicb.2023.1176582)
Supplement: Supplementary file 2 [file Data_Sheet_1.pdf]

SUPPLEMENTARY INFORMATION FOR:

Bacterial molecular machinery in the Martian cryosphere conditions

Víctor Muñoz-Hisado[1#], Fátima Ruiz-Blas[2#], Jesús Manuel Sobrado[1], Eva Garcia-Lopez[1], Emma Martinez-Alonso[3], Alberto Alcázar[3] and Cristina Cid[1]*

1 Centro de Astrobiologia (CAB), CSIC-INTA, Carretera de Ajalvir km4, 28850 Torrejón de Ardoz, Madrid, Spain.

2 GFZ German Research Centre for Geosciences, Section Geomicrobiology, Telegrafenberg, 14473 Potsdam, Germany

3 Hospital Ramón y Cajal, Instituto Ramón y Cajal de Investigación Sanitaria, 28034 Madrid, Spain.

# These authors contributed equally to this work.

*Correspondence: Cristina Cid, Centro de Astrobiología (CSIC-INTA), Ctra. Ajalvir, km 4, 28850 Torrejón de Ardoz, Madrid, Spain. cidsc@inta.es; cidsc@cab.inta-csic.es; Tel.: +34 91 520 5455.

This file includes:

I. Supplementary Tables. S1.

II. Supplementary Figures. S1-S3.

III. Data availability statement

I. Supplementary Tables

Table S1. Mass spectra of *Bacillus subtilis* and *Curtobacterium flacumfaciens* samples
File: Table_S1.xlsx.

II. Supplementary Figures.

Figure S1. Growth curves of Bacillus and Curtobacterium. The data were the mean ± SD from three replicates.

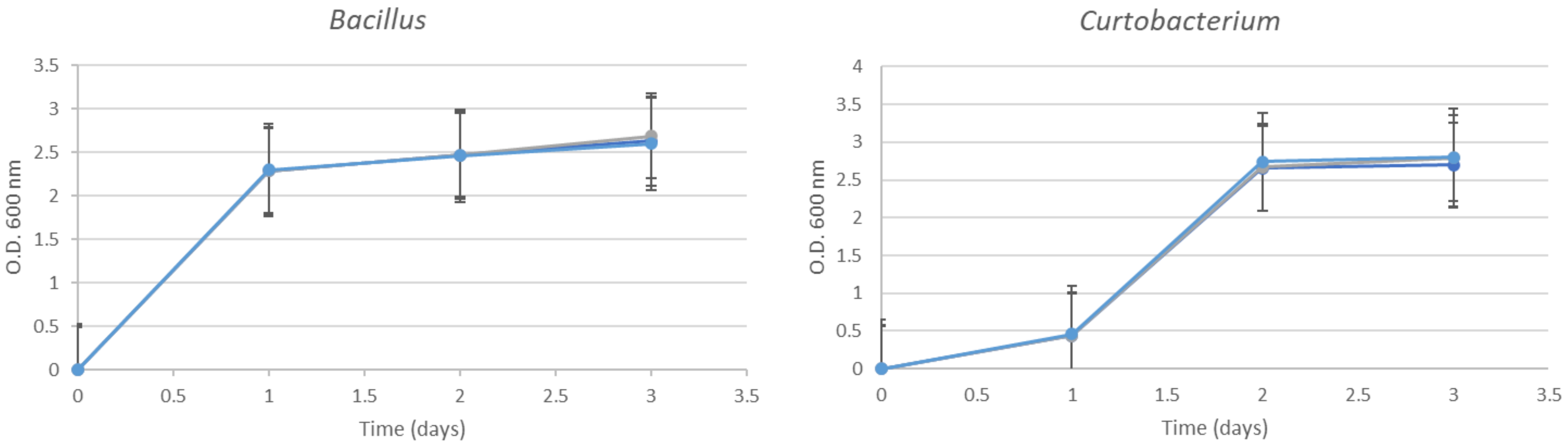

Figure S2. Survival fraction of bacteria before and after exposure to Martian conditions in the MARTE chamber. (A) *Bacillus subtilis*. A: before, AM: after. (B) *Curtobacterium flacumfaciens*. B: before, BM: after. Statistical differences were studied by Student's t test (***, $p < 0.0001$).

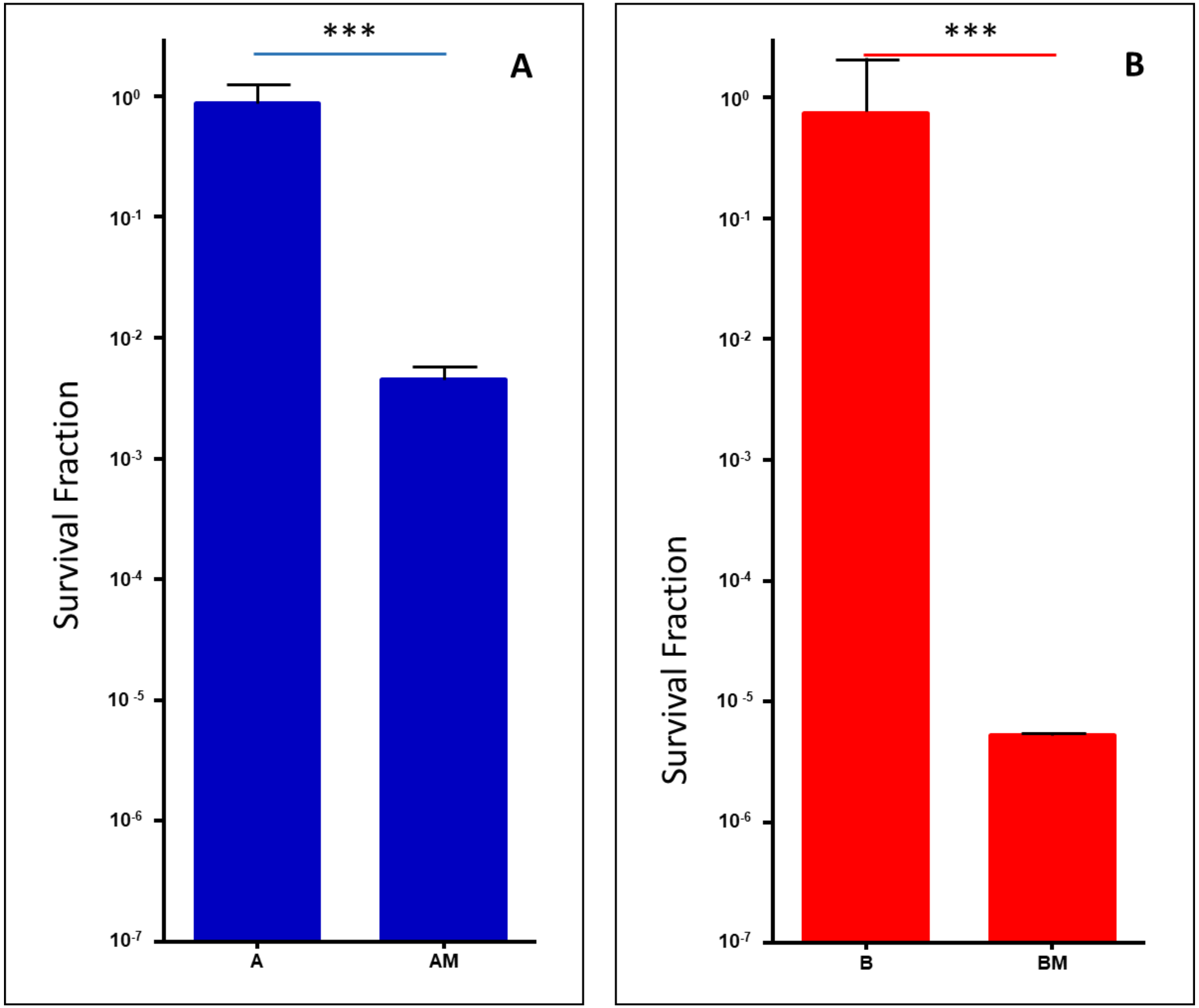

Figure S3. Images of 2D-DIGE gels. 2D-DIGE gels of (A) *Bacillus subtilis* and (B) *Curtobacterium flacumfaciens* protein extracts from cultures under standard and Martian conditions. Images show the overlay of samples from standard conditions labelled with Cy3 fluorochrome and samples from Martian conditions labelled with Cy5 fluorochrome, showing the differences in the expression levels of the protein spots that appear as a result of exciting both fluorochromes at the same time.

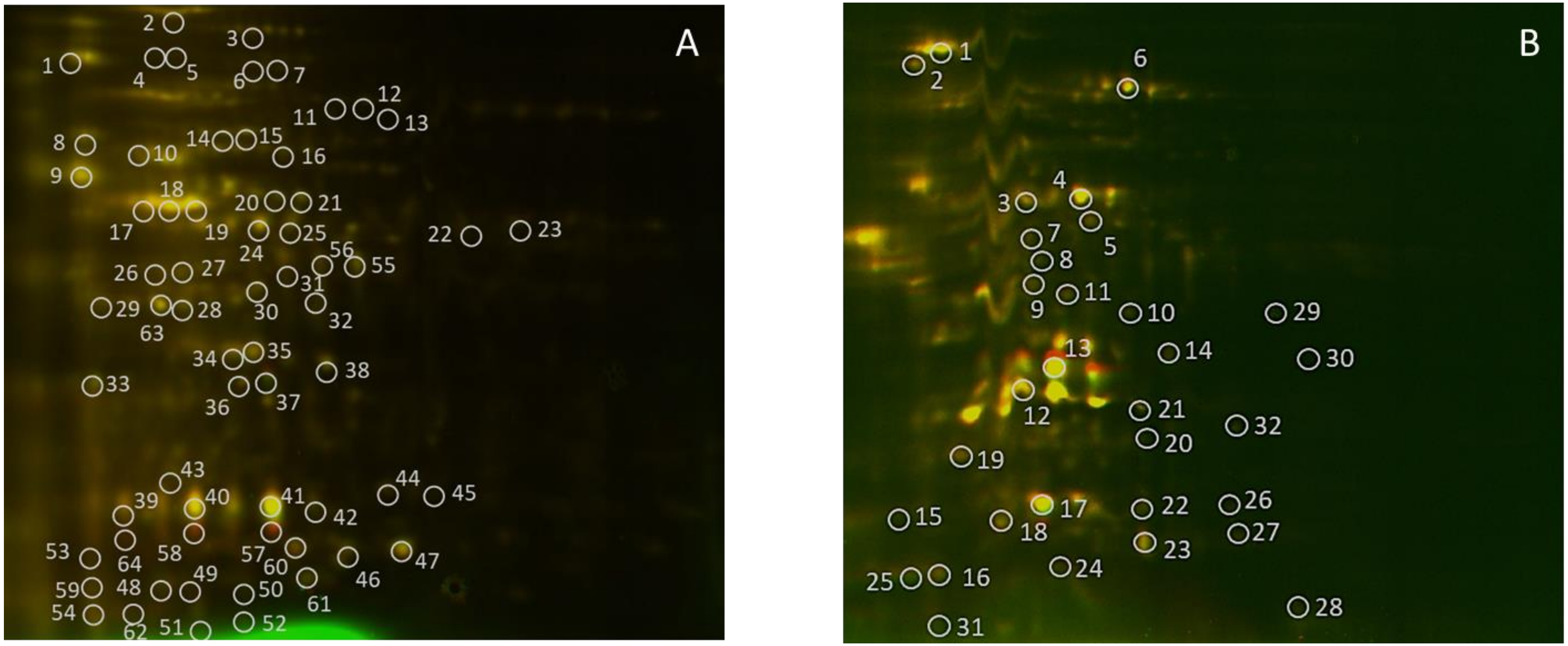

III. Data availability statement

The mass spectrometry proteomics data are deposited to the ProteomeXchange Consortium via the PRIDE repository with the dataset identifiers PXD027245 (samples A and AM) and PXD027249 (samples B and BM).
